# Death Receptor 5 Displayed on Extracellular Vesicles Decreases TRAIL Sensitivity of Colon Cancer Cells

**DOI:** 10.3389/fcell.2020.00318

**Published:** 2020-05-19

**Authors:** Rita Setroikromo, Baojie Zhang, Carlos R. Reis, Rima H. Mistry, Wim J. Quax

**Affiliations:** Department of Chemical and Pharmaceutical Biology, Groningen Research Institute of Pharmacy, University of Groningen, Groningen, Netherlands

**Keywords:** extracellular vesicles, DR5, TRAIL, apoptosis, conditioned medium, receptor–ligand trafficking

## Abstract

Tumor necrosis factor–related apoptosis inducing ligand (TRAIL) is considered to be a promising antitumor drug because of its selective proapoptotic properties on tumor cells. However, the clinical application of TRAIL is until now limited because of the resistance of several cancer cells, which can occur at various levels in the TRAIL signaling pathway. The role of decoy receptors that can side-track TRAIL, thereby preventing the formation of an activated death receptor, has been extensively studied. In this study, we have focused on extracellular vesicles (EVs) that are known to play a role in cell-to-cell communication and that can be released by donor cells into the medium transferring their components to recipient cells. TRAIL-induced apoptotic signaling is triggered upon the binding of two death receptors, DR4 and DR5. Here, we found that DR5 but not DR4 is present in the conditioned medium (CM)–derived from various cancer cells. Moreover, we observed that DR5 was exposed on EVs and can act as “decoy receptor” for binding to TRAIL. This results in a strongly reduced number of apoptotic cells upon treatment with DR5-specific TRAIL variant DHER in CM. This reduction happened with EVs containing either the long or short isoform of DR5. Taken together, we demonstrated that colon rectal tumor cells can secrete DR5-coated EVs, and this can cause TRAIL resistance. This is to our knowledge a novel finding and provides new insights into understanding TRAIL sensitivity.

## Introduction

The secretion of extracellular vesicles (EVs) is an evolutionally conserved process spanning from bacteria to humans and plants (Rivera et al., 2010; Van Niel et al., 2018; Cui et al., 2019). The significance of EVs on the one hand relates to their capacity to eliminate unwanted components from the cell and on the other hand to their capability to communicate with other cells by exchanging components–from DNA to protein–and thereby influencing the signal transduction pathways of target cells (Colombo et al., 2014; Yáñez-Mó et al., 2015). They are highly heterogeneous and can be broadly divided into two main categories based on their biogenesis and characterizations (Colombo et al., 2014; Van Niel et al., 2018). The term *exosomes* (30–100 nm) was first used to describe the EVs released by reticulocytes during differentiation (Johnstone et al., 1987). It originates from inward budding of endosome membrane creating the so-called cargo-containing intraluminal vesicle (ILV) inside the early endosome. These early endosomes can either be directed to the lysosomes or fused together and mature to the late multivesicular endosomes (MVEs). MVEs when fused with cell membrane can release their cargo-containing ILV in the extracellular space, and these small vesicles are called exosomes (McGough and Vincent, 2016). The other group of EVs is named microvesicles (50–1,000 nm, up to 10 μm), which are directly formed after budding or fission of plasma membrane in response to diverse cell stimulation; this includes the apoptotic bodies. Owing to their varied compositions, increasing evidence shows that EVs act as signaling vesicles not only in normal cell homeostasis but also in many pathological conditions (Cocucci et al., 2009).

Cancer is a diverse group of diseases caused by proliferating cells traditionally treated with chemotherapy and/or radiotherapy. These, however, also give harmful side effects to healthy cells. More preferred therapeutics are being developed in such a way that they selectively target cancer cells and treatment with tumor necrosis factor–related apoptosis inducing ligand (TRAIL) is considered to be promising because of its naturally proapoptotic properties specifically directed to cancer cells (Wong et al., 2019). Binding of TRAIL to two death receptors (DR4 and DR5) triggers the recruitment of Fas-associated death domain and subsequent pro–caspase-8. This complex, also known as death-inducing signaling complex (DISC), will initiate downstream caspase-dependent apoptotic signaling and eventually leads to cell death (Nagata, 1997). Although cancer cells are more prone to TRAIL-induced cell death than normal cells, this signaling pathway can be interrupted by many other factors that lead to resistance in several cancer cells. For instance, three decoy receptors (DcR1, DcR2, and OPG) can also bind to TRAIL and thereby decrease the availability of free TRAIL for the binding to the death receptors, leading to inhibition of apoptosis (Mahalingam et al., 2009). Despite the importance of this classical ligand–receptor binding to induce apoptosis, ligand-induced receptor internalization, and/or intercellular receptor trafficking are also important for adequate transduction of the apoptosis signaling. Likewise, nuclear localization of DR5 by importin β1 decreases TRAIL-induced cell death in human tumor cells (Kojima et al., 2011). The presence of death receptors in autophagosomes rather than plasma prevents TRAIL-induced apoptosis in breast cancer cells (Di et al., 2013). In addition, the surface levels of DR4 are controlled by MARCH-8–mediated ubiquitination, which results in differential endosomal trafficking of surface DR4 and DR5, and thereby regulates the resistance to TRAIL (Van De Kooij et al., 2013). Given the evidences that degradation and secretion of death receptors are important for the extent of the apoptosis signaling, we want to know if death receptors are secreted and expressed on the surface of EVs.

In this study, we demonstrate that DR5 molecules are on the surface of EVs, and these can compete with the DR5 on target cells for TRAIL binding, leading to a decrease of the apoptosis signaling. These findings contribute a new insight into mechanisms of TRAIL resistance.

## Materials and Methods

### Cell Lines and Culture Conditions

Human colorectal carcinoma cell lines (Colo205, HCT 116, and DLD-1), human Burkitt lymphoma B cell line (BJAB), and the Chinese hamster ovary cell line (CHO) were cultured in RPMI1640 medium supplemented with 10% fetal bovine serum, 100 U/mL penicillin, and 100 μg/mL streptomycin in a humidified incubator at 37°C with 5% CO_2_. All materials mentioned above were purchased from Thermo Fisher Scientific (Waltham, MA, United States). BJAB cell lines, the wild-type cells BJAB (BJAB WT), BJAB overexpressing DR5 (BJAB DR5), and a deficient DR5 short isoform (BJAB DR5s DEF) were kindly provided by Dr. Andrew Thornburn (University of Colorado Health Sciences Centre, Aurora, CO, United States). CHO cell lines, the wild-type cells (CHO WT), a mutant overexpressing DR5 long isoform (CHO TV1), and a mutant overexpressing DR5 short isoform (CHO TV2) were provided by Organon (Oss, Netherlands).

### Reagents

Soluble (aa 114–281) wild-type TRAIL (TRAIL WT), DR4-specific TRAIL variant (4C7), and DR5-specific TRAIL variant (DHER) were constructed and produced as previously described (Van Der Sloot et al., 2006; Reis et al., 2010).

### Collecting Conditioned Medium and Isolation of EVs

Cells were cultured at the concentration of 150,000 cells/mL in exosome-free medium for 48 h in humidified incubator at 37°C with 5% carbon dioxide. Medium was collected and spun down at 250 *g* for 10 min to discard the floating cells. This supernatant is from now on called conditioned medium (CM). EVs were isolated by differential centrifugation strategy: first, sedimentation of CM at 3,000 *g* for 15 min; second, sedimentation of the supernatant at 17,000 *g* for 20 min; and finally with ultracentrifugation at 30,000 *g* for 3 h. From the last run, the pellet was used as EVs and resuspended in phosphate-buffered saline (PBS) and stored at −80°C.

### Cell Viability Assay

Cell viability assays were conducted using MTS assay. Cells were seeded in triplicate in 96-well plates at the density of 10,000 cells/mL in medium and incubated in a humidified incubator at 37°C with 5% CO_2_. The following day, cells were treated with TRAIL WT or variants for 24 h, and assayed for viability with MTS reagent according to the manufacturer’s instruction (Promega, Madison, WI, United States). The cell viability was determined by measuring the absorbance at 490 nm using a microplate reader (Thermo Labsystems, Helsinki, Finland).

### Western Blot

Cells were harvested and lysed with RIPA buffer supplemented with EDTA-free proteinase inhibitor cocktail (Roche, Basel, Switzerland). Samples were loaded on precast 4 to 12% sodium dodecyl sulfate–polyacrylamide gel electrophoresis gels (Thermo Fisher Scientific) and transferred onto 0.45 μm nitrocellulose membrane. Next, the membranes were blocked for 1 h at room temperature in 5% non-fat milk and probed overnight at 4°C. The following primary antibodies were used: DR5 (Sigma, Zwijndrecht, Netherlands), DR4 (Imgenix, Cambridge, United Kingdom), histone H2A (Abcam, Cambridge, United Kingdom), and CD63 (Pharmingen, San Diego, CA, United States). After incubating with secondary antibodies, membranes were detected using Pierce ECL kit (Thermo Fisher Scientific).

### Apoptotic Assay

Apoptosis induction was measured using annexin V–fluorescein isothiocyanate (FITC) staining and quantified by flow cytometry. Cells were seeded in six-well plates overnight prior to the treatment. The next day, cells were treated with TRAIL variant for 24 h. After treatment, cells were collected, washed with PBS twice, and incubated for 20 min with annexin V–FITC solution on ice. The cells were analyzed using a FACS Calibur flow cytometer (BD Biosciences, Franklin Lakes, NJ, United States).

### Detection of DRs on EVs by Transmission Electron Microscopy

The isolated EV suspension was incubated with DR5 antibody (ENZO life sciences, Bruxelles, Belgium) and placed as a drop gently on formvar/carbon-coated nickel grid for 60 min. The grids were washed three times with 0.1% exosome-free bovine serum albumin PBS solution and incubated for 10 min in 2% paraformaldehyde. The grids were washed three times with PBS and incubated for 40 min with secondary antibody conjugated with 10-nm gold particles. The grids were washed 3 times with PBS and post fixated with 2.5% glutaraldehyde for 10 min and 2% uranyl acetate for 15 min. The excess liquid was gently removed from the grids and dried before analyzing under transmission electron microscope.

### Data Analysis

Data are presented as mean ± SD from triplicates in one experiment, and experiments were repeated three times. *P* values were analyzed by two-way analysis of variance in Tukey multiple comparisons with GraphPad Prism version 7.0 (San Diego, CA, United States). ^∗∗^*p* ≤ 0.01, ^∗∗∗^ *p* ≤ 0.001, and ^****^p ≤ 0.0001. Data from apoptosis assays were analyzed by FlowJo V10 (BD Biosciences).

## Results

### Conditioned Medium Inhibits DR5-Mediated Cell Death in Cancer Cells

**FIGURE 1:**
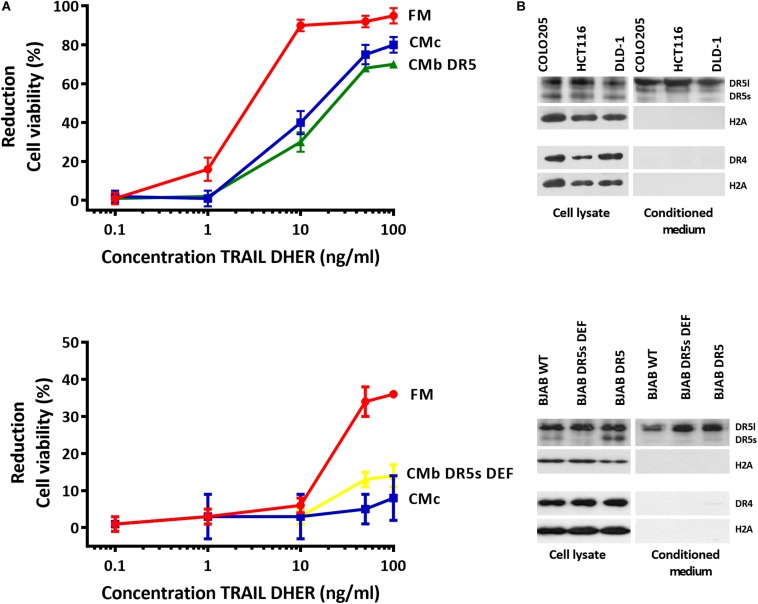
Conditioned medium inhibits DR5-mediated apoptosis in Colo205 cells. Colo205 (**A**, upper) and BJAB cells (**A**, below) were treated with TRAIL DHER variant for 24 h in the presence of fresh medium (FM) or conditioned medium derived from either Colo205 (CMc), BJAB expressing both DR5 isotypes (CMb DR5), or BJAB cells deficient for DR5 short isoform (CMb DR5s DEF) cells. Conditioned medium was collected after cultivation of cells at a density of 150,000 cells/mL for 48 h. Cell death was measured by MTS assay. Data expressed as the mean ± SD of triplicate samples. Similar results were obtained in three independent experiments. **(B)** Total cell extract and conditioned medium (CM) of three colon carcinoma cell lines (upper) and Burkitt lymphoma cell lines (below) were analyzed for DR4, DR5, and H2A expression with Western blot. The absence of H2A in CM indicates no contamination of cellular nucleosome proteins. Similar results were obtained in three independent experiments.

Most cancer cells release EVs, and the mode of action of those organelles depends on their cargo proteins (Raposo et al., 1996; Denzer et al., 2000; Rivoltini et al., 2016). We hypothesize that secreted death receptors displayed via the EVs can act as decoy receptors and therefore reduce the apoptosis signaling. We cultivated Colo205 and BJAB cells at the concentration of 150,000 cells/mL in exosome-free medium for 48 h, and the medium was collected and used as CM. Colo205 and BJAB cells were treated for 24 h with DR5 TRAIL variant (DHER) in fresh medium or CM derived either from Colo205 (CMc) or BJAB (CMb). We examined the cell viability of Colo205 and BJAB cells with MTS assay. We also used in this experiment BJAB mutants that expressed respectively only the DR5 long isoform (CMb DR5s DEF) or both isoforms (CMb DR5). We observed in both TRAIL-treated cells incubated in fresh medium considerable higher percentages of cell death than in cells grown in CM. This protection was observed for CM derived from Colo205 as from BJAB cells. The protective effect of CM was dose dependent and most prominent at 10 ng/mL DHER for Colo205 and for BJAB DR5 cells at 50 ng/mL (Figure 1A). This indicates that the CM contains factors that are able to inhibit DR5-mediated cell death signaling. Interestingly, Western blot analysis of the CM from three different colon carcinoma cells (Colo2015, HCT116, and DLD-1) and BJAB mutants revealed that only DR5 was secreted in significant levels in CM, and DR4 levels were almost negligible (Figure 1B). Next, the long DR5 isoform seems to be sufficient for this protective effect as cells expressing only the long isoform (BJAB DR5s DEF) were also able to reduce the cell death. The absence of H2A in the supernatant confirms the purity of the sample preparation and absence of cellular nucleosome proteins in the CM.

### Both Long and Short Isoforms of DR5 in CM Contribute to TRAIL Resistance

**FIGURE 2:**
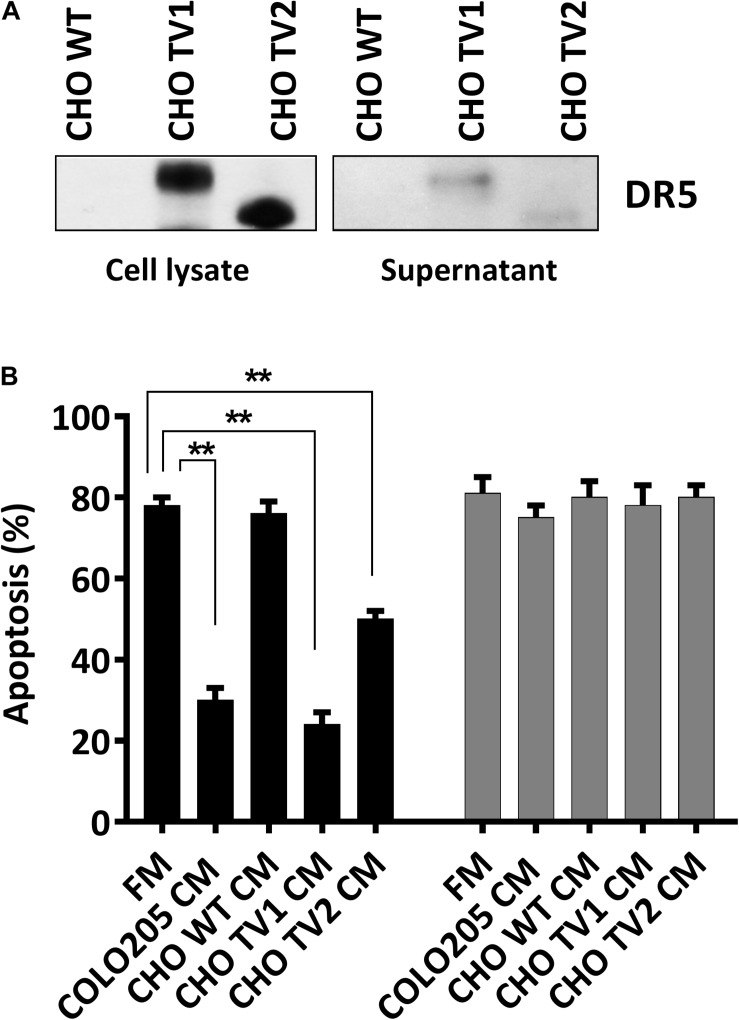
Long and short isoforms of DR5 in conditioned medium protect against DR5-mediated apoptosis. **(A)** Western blot analysis of the expression of DR5 long and short isoforms of CHO wild-type (WT), CHOTV1, and CHO TV2 mutants in total cell lysate or in conditioned medium (CM). **(B)** Colo205 cells were treated with 10 ng/mL DHER (black) or 4C7 (gray) TRAIL variants for 24 h in the presence of fresh medium or conditioned medium derivate from either Colo205, CHO TV1, or CHO TV2 cells. Apoptosis was measured by annexin V staining. Data expressed as the mean ± SD of triplicate samples. Similar results were obtained in three independent experiments.

We have concluded that CM can prevent DR5-mediated cell death. To explore which isoforms of DR5 contribute to this resistance phenomenon, we used CHO cells expressing either the human long, or the short DR5 isoforms. Immunostaining with DR5 antibodies confirmed the expression of the different DR5 isotypes in total cell lysates of CHO mutants (CHO-TV1 and CHO-TV2), and both isotypes were secreted into the CM (Figure 2A). Treatment of Colo205 cells with 10 ng/mL TRAIL DHER variant in CM derived from Colo205, CHO-TV1, or CHO-TV2 cells resulted in significant inhibition of apoptosis compared to fresh medium or CM derived from CHO wild-type cells (CHO-WT CM), which lack both DR5 isoforms. This protective effect was specifically related to DR5, as no protection was observed with the 4C7 variant, which can induce apoptosis only via DR4 receptor (Figure 2B). The protective effect of CHO-TV1–derived CM versus CHO-WT–derived CM was at the same magnitude as COLO205-derived CM versus fresh medium. The short isoform (CHO-TV2) showed a slightly lower protective effect. We were not able to quantify the precise concentration of DR5 in the CMs, and therefore we only can conclude that both long and short isoforms of DR5 contribute to TRAIL-resistance mechanism of Colo205 cells.

### DR5 Is Expressed at the Surface of EVs

**FIGURE 3:**
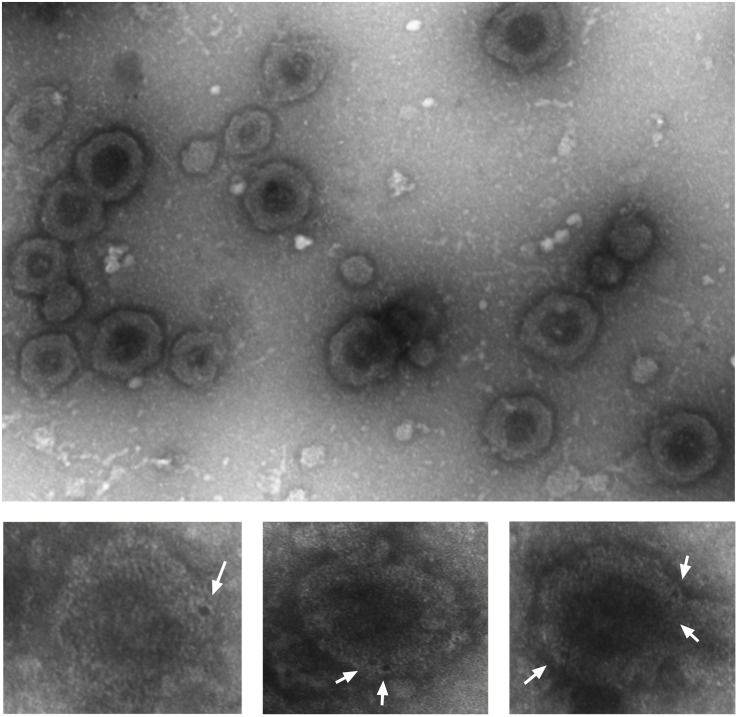
Colo205 conditioned medium contains DR5-coated extracellular vesicles. Negative staining of EVs isolated from conditioned medium (upper picture). Bottom pictures are the zoom pictures of single EV stained with gold-labeled DR5 antibody (white arrows) and detected by transmission electron microscope. Scale bar is 500 nm. The experiment was repeated three times, and several EVs were analyzed.

To investigate whether DR5 was secreted out of the cells as soluble receptors or packed into vesicles, we fractionated the CM by differential centrifugation strategy and analyzed it with transmission electron microscope. The smallest vesicles ranging from 30 to 300 nm were sedimented by ultracentrifugation at 100,000 *g*. Bigger particles were first removed stepwise at lower speeds to avoid artificial small vesicles formation (Livshits et al., 2016). After negative staining various exosome-like vesicles, characteristics such as donut-like structures with different sizes, and shapes were observed (Figure 3, upper picture). Next, we asked whether secreted EVs are coated with DR5. Immunostaining with gold-labeled DR5 antibody showed DR5 at the surface of the EVs, visible as dark spots at the surface of the EV (Figure 3, bottom pictures).

### Depletion of EVs in CM Restores the TRAIL DHER Sensitivity of Colo205 Cells

**FIGURE 4:**
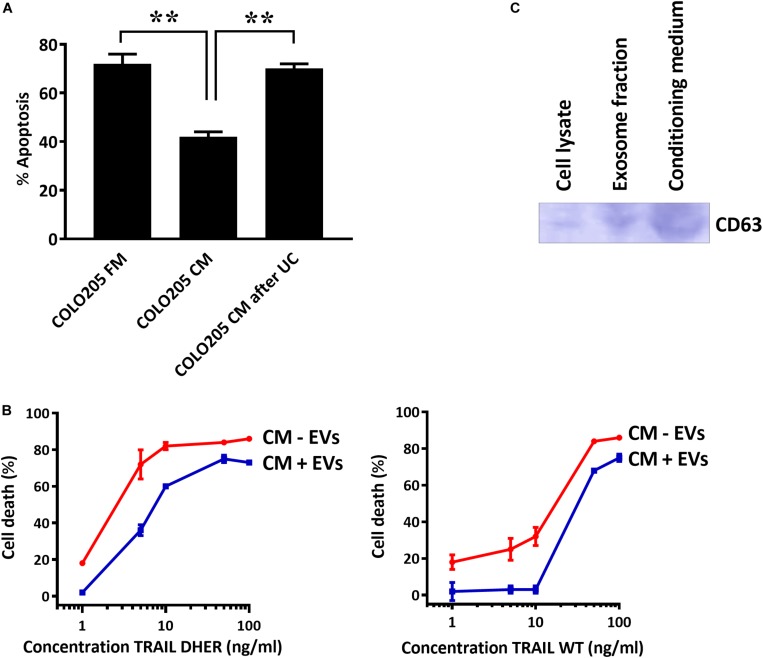
DR5-coated EVs inhibit TRAIL-induced apoptosis. **(A)** Colo205 cells treated with TRAIL DHER in fresh medium (FM), conditioned medium (CM), or conditioned medium after ultracentrifuging (UC) and apoptotic cells were determined by annexin V staining. **(B)** Colo205 cells were cultured in CM containing either EVs (CM + EV) or depleted of EVs (CM - EV) and followed by the treatment with TRAIL DHER or TRAIL wild type (WT). Cell death was measured by MTS assay. **(C)** Western blot of CD63. Data expressed as the mean ± SD of triplicate samples. Similar results were obtained in three independent experiments.

To confirm that the secreted EVs coated DR5 are responsible for the protection against cell death, we depleted EVs from CM and treated the cells with TRAIL DHER. Removing EVs from CM by sedimentation nullified completely the protective effect of CM upon TRAIL DHER treatment in Colo205 (Figure 4A). This protective effect was again observed when purified EVs were supplemented to fresh medium in Colo205 cells treated with TRAIL DHER or TRAIL wild type (Figure 4B). CD63 was used as positive control for the isolation of EVs (Figure 4C).

## Discussion

In the present study, we showed EVs coated with DR5 receptors can reduce the TRAIL-mediated apoptosis in cancer cells. This inhibition of the EVs was specific when apoptosis is triggered by DR5. TRAIL 4C7 variant, which triggers apoptosis via DR4, was not inhibited by CM. Both long and short isoforms of DR5 contribute to the inhibition of TRAIL-mediated apoptosis. This is the first report demonstrating the expression of DR5 on the surface of EVs, providing a new insight into the TRAIL-resistance phenomenon.

The endocytosis of TRAIL-DR complex and its importance on triggering the apoptosis signaling have been studied extensively. However, there are conflicting reports as to whether internalization of TRAIL-DR complex results either in inhibiting or enhancing the apoptotic signals depending on the cell types (Austin et al., 2006; Kohlhaas et al., 2007; Reis et al., 2017). One study showed that DR-mediated caspase activation rapidly disrupts clathrin-mediated endocytosis (CME), which in turn enhanced the apoptotic signals downstream of the DISC complexs (Austin et al., 2006). Recently, another study unraveled the molecular mechanism of CME-dependent endocytosis of death receptors. They showed that endocytosis of TRAIL-DR complex requires dynamin-1 protein, which is activated by ryanodine receptor-mediated Ca^2+^ release in response to caspase-8 activation. However, this selective regulation of TRAIL-DR endocytosis suppresses TRAIL-mediated apoptosis (Reis et al., 2017).

Internalized receptor complexes in the endocytic pathway can undergo different routes: receptors can be processed and recycled back to the surface or enter the degradation machinery. Ubiquitination of ligand–receptor complexes plays an important role in the endosomal sorting mechanism into MVE to direct the cargo toward the degradation machinery and in this way determine the fate of the protein. A study reported that the membrane-associated RING-CH ubiquitin ligase 8 (March-8) regulates the cell surface expression of DR4 and targets DR4 to the lysosomal degradation machinery (Van De Kooij et al., 2013). Interesting in their study was that March-8 had noticeable less preference for targeting DR5. Lys-273 at the cytoplasmic tail of DR4 is an important ubiquitin acceptor sites for March-8, and DR5 has no Lys-273 residue or homolog at membrane-proximal locations. Therefore, inefficient targeting of DR5 to lysosomes may be the reason that DR5 is preferentially displayed at EVs. Apart from internalization of receptors, receptors can also be released in the medium by exocytosis. This involves the release of small vesicle-like structure, which carries biomolecules such as plasma membrane receptors and other proteins into the extracellular space. The effect of the secreted DR-coated EVs on the apoptosis signaling has hardly been studied and may explain the variation in TRAIL response of cancer cells. Proteomic database search in Vesiclepedia^1^ revealed that DR5 is present in exosomes of several cancer cells from brain, colorectal, kidney, glioblastoma, ovarian, prostate, lung, leukemia, and melanoma cancer. However, no functional biological data exist on the influence of DRs on EVs on TRAIL sensitivity. Despite the interesting findings of differential endocytosis and ubiquitination of DRs, more research should be done to understand the mechanism of intracellular receptors trafficking. Together with the new insight in TRAIL-resistance mechanism by DR5-coated EVs, TRAIL treatment in combination with inhibitors preventing secretion of EVs could be a promising combination strategy to treat TRAIL-resistant cancer cells.

In summary, we have uncovered the role of DR5-coated EVs in the resistance of cancer cells for TRAIL treatment. Secreted DR5-coated EVs inhibit TRAIL sensitivity of colon cancer cells. This protective effect was specific for DR5, as DR4 was absent in CM.

## Data Availability Statement

All datasets generated for this study are included in the article/supplementary material.

## Author Contributions

WQ is the principal investigation. CR initiated the concept of the manuscript. CR, RS, and BZ designed the experiments. CR, RS, BZ, and RM performed the experiments and analyzed the data. The manuscript was written by RS and BZ and was carefully revised by WQ.

## Conflict of Interest

The authors declare that the research was conducted in the absence of any commercial or financial relationships that could be construed as a potential conflict of interest.

## Footnotes

1 http://microvesicles.org
